# MVA-BN monkeypox vaccine safety in a clinical trial in the Democratic Republic of the Congo

**DOI:** 10.1038/s41541-026-01490-0

**Published:** 2026-05-28

**Authors:** Nsengi Ntamabyaliro, Tyler Bonnett, Gail Potter, Emily Yox, Laurens Liesenborghs, Olivier Tshiani Mbaya, Veronique Nussenblatt, Tarcisse Kapene Kilara, Aline Biongo Engo, Emmanuella Muhima, Percy Velela, Laetitia Itunime, Jean-Luc Biampata, Placide Mbala, Mariano Lusakibanza, Michael Kombozi Basika, Gaston Tona, Susan Vogel, H. Clifford Lane, Lori E. Dodd, Jean-Jaques Muyembe

**Affiliations:** 1 Clinical Pharmacology and Pharmacovigilance Unit, Faculty of Medicine, University of Kinshasa, Kinshasa, Democratic Republic of the Congo; 2 Institut National de Recherche Biomédicale, INRB, Kinshasa, Democratic Republic of the Congo; 3 Clinical Monitoring Research Program Directorate, Frederick National Laboratory for Cancer Research, Frederick, MD USA; 4 Clinical Trials Research and Statistics Branch, Office of Biostatistics Research, National Institute of Allergy and Infectious Diseases, National Institutes of Health, Bethesda, MD USA; 5 Department of Clinical Sciences, Institute of Tropical Medicine, Antwerp, Belgium; 6 Department of Microbiology, Immunology and Transplantation, KU Leuven, Leuven, Belgium; 7 Laboratory of Clinical Immunology and Microbiology, National Institute of Allergy and Infectious Diseases, National Institutes of Health, Bethesda, MD USA

**Keywords:** Diseases, Health care, Medical research

## Abstract

The MVA-BN vaccine is considered safe and effective and has been widely deployed to prevent monkeypox. However, safety and tolerability data from endemic areas are limited. We performed a single-arm clinical trial of the safety of the MVA-BN vaccine among adults in the Democratic Republic of the Congo (ClinicalTrials.gov: NCT05734508; Registered 21 February 2023). Participants were personnel working on a monkeypox therapeutics trial (PALM-007) conducted in a high-risk, endemic setting for monkeypox. Participants received two doses of vaccine 28 days apart and were actively followed up to 28 days after each dose to assess the safety profile of MVA-BN. From March 2023 to June 2024, 500 participants were enrolled and received their first vaccine dose; 494 (99%) received the second dose and associated follow-up. There were no adverse events (AEs) of grade 3 or higher observed during the study. 175 participants (35%) reported at least one vaccination site AE, the most common of which was mild or moderate vaccination site pain (120/500 first dose recipients (24%); 48/494 second dose recipients (10%)). There was one recorded case of breakthrough monkeypox occurring approximately 3 months after the second dose. This observational study provides further evidence that the MVA-BN monkeypox vaccine is generally safe and well-tolerated. As previously reported, the most common AEs were mild or moderate injection site reactions.

## Introduction

Monkeypox is an emerging viral disease that, due to its rapid geographic expansion, has been considered a Public Health Emergency of International Concern (PHEIC) by the World Health Organization (WHO). The disease, characterized by its typical rash, is caused by the monkeypox virus (MPXV), of which two genetically distinct clades are known: Clade I, which is endemic in Central Africa, and Clade II, which is endemic in West Africa and was responsible for a recent global outbreak in 2022^1^.

The first human case of monkeypox occurred in a 9-year-old boy, likely infected through contact with monkey meat, in 1970 in the Democratic Republic of the Congo (DRC)^2^. Most subsequent cases, caused mainly by Clade I MPXV infection, have been reported from tropical forest areas of Central Africa. Historically, the majority of monkeypox outbreaks have been caused by the zoonotic spillover of Clade I MPXV in rural areas of Central Africa, particularly in the DRC^1^. In these settings, humans can acquire infection through direct contact with infected animals, often while hunting, trapping, and/or processing infected animals, or their body parts and fluids^2^. In contrast, Clade II MPXV is endemic to West Africa and usually causes milder disease linked to animal spillovers^3,4^. Both clades can spread through human contact, but Clade I rarely transmits beyond initial cases, while Clade II—especially IIb—has shown greater sustained human-to-human transmission, as seen in the 2022 global outbreak^5,6^

A major shift in transmission dynamics occurred in 2022, when a subclade of Clade II, designated Clade IIb, began spreading through sustained human-to-human transmission, primarily through sexual contact^5^. This resulted in the international spread of Clade IIb, which caused over 100,000 infections globally, mainly in Europe, the Americas, and Southeast Asia^6–9^ and prompted the WHO to declare a PHEIC in July 2022^10^.

By 2023, the Clade IIb outbreak had largely subsided thanks to a combination of targeted vaccination campaigns, increased awareness, and behavioral interventions^7,8^. However, following this decline in Clade IIb cases, the epicenter shifted again to Central Africa and the DRC. In September 2023, a distinct lineage within Clade I, referred to as Clade Ib, was identified in the eastern part of DRC. This subclade has been associated with sustained human-to-human transmission and an increase in reported cases in the region, including spread to bordering countries^9,11^. This contributed to a renewed international concern^12^ that led to a second PHEIC declaration in August 2024^10^.

Large-scale vaccination is one of the most important strategies to curb the further spread of monkeypox. The most commonly used vaccine is the Modified Vaccinia Ankara-Bavarian Nordic (MVA-BN), an attenuated, live, non-replicating virus. MVA-BN was developed to protect against variola virus, the causative agent of smallpox, but also cross-protects against MPXV in non-human primate models. The vaccine is administered subcutaneously or intradermally in two doses separated by at least 28 days^13,14^.

Previous studies have indicated that MVA-BN is generally safe, although limited results are available from cohorts in DRC, where the disease burden is highest. The present study is the second to report safety results in a Congolese cohort, following the very recent series of reports by Minhaj et al.^15^ and Priyamvada et al.^16^, that reported safety results for the same large, prospective cohort study of vaccinated healthcare workers in DRC, some of whom later received a booster dose and provided long-term immunogenicity data (anti-orthopoxvirus IgM and IgG). Notably, the series of studies and the present study were all carried out among healthcare workers. The most frequently reported adverse events (AEs) associated with MVA-BN vaccination are mild injection site events, such as injection site pain and injection site induration^17–19^.

MVA-BN was approved by regulatory authorities in the USA (JYNNEOS) in September 2019^20^ and Europe (Imvanex) in July 2022^21^ for the prevention of MPXV infection. In July 2022, the DRC pharmaceutical regulatory authority, ACOREP (*Autorité Congolaise de Réglementation Pharmaceutique*), reviewed the efficacy and safety profile of MVA-BN in previous studies and approved its use to protect staff of an ongoing monkeypox therapeutics randomized controlled trial in DRC (PALM 007), with the requirement for active collection and reporting of AEs to assess the safety profile of the vaccine in a cohort of Congolese participants for the first time. Here we describe the results of the ensuing study of MVA-BN safety, conducted at three sites in the DRC from March 2023 to June 2024.

## Methods

### Study Design

This paper reports the results of PALM 008 (ClinicalTrials.gov: NCT05734508; Registered 21 February 2023), a single-arm clinical trial of the safety profile of the MVA-BN monkeypox vaccine in the DRC. The study was conducted at three sites: the Tunda and Kole General Hospitals, in Maniema and Sankuru provinces, DRC, and the main campus of the INRB (*Institut National de Recherche Biomédicale*) in Kinshasa, DRC. Adult participants (≥18 years) were voluntarily recruited from PALM 007 study staff, and all participants provided informed consent prior to engaging in any study procedures. Exclusion criteria included (1) pregnant or breastfeeding women, (2) individuals with fever (≥37.5°C), and (3) any individual who had previously received the MVA-BN vaccine.

### Trial oversight and conduct

The protocol was approved by the Ethics Committee of the University of Kinshasa School of Public Health and ACOREP. The trial was supervised locally under the auspices of the INRB and the National Pharmacovigilance Center of the DRC in accordance with ICH GCP-E6 R2 at the PALM 007 study sites. Local staff were trained on study procedures with oversight provided by INRB staff.

### Trial procedures and safety assessments

Informed consent was obtained from all enrolled subjects prior to subcutaneous administration of 0.5 mL MVA-BN (in either shoulder) as two shots given 28 days apart. Participants remained under observation for 30 minutes following each shot, then completed, either in-person or by phone, follow-up visits at days 3, 14, and 28 post-vaccination. Prior to the first vaccine, a medical history was obtained, including (1) history of orthopox virus disease, (2) current comorbidities, and (3) medications. A clinical examination was carried out for all participants. A pregnancy test was performed for women of childbearing potential prior to each vaccine administration. Participants detected as pregnant while included in the study were not allowed to receive further doses but remained under follow-up until the outcome of their pregnancy could be ascertained.

During the 30-minute observation window and each follow-up visit, participants were asked to report AEs via open-ended questions. The severity of each AE was graded using Common Terminology Criteria for Adverse Events (CTCAE) version 5.0^22^, a broadly used and well-known set of standardized grading criteria. AEs were coded using the Medical Dictionary for Regulatory Activities (MedDRA) version 25.1 or higher. No formal causality assessment between AEs and the vaccine was performed. All AEs temporally associated with vaccination were recorded and reported without attribution.

The primary objectives were to estimate the incidence of serious adverse events (SAEs) in all vaccinated individuals and to report the proportion of participants experiencing at least one AE within 14 days after the first vaccine dose and within 28 days after the second dose. SAEs were defined according to the International Conference on Harmonization guidelines^23^.

### Sample size and statistical analysis

The protocol allowed for a sample size of up to 1000 participants, with the goal being to obtain the largest sample possible given the descriptive goals of the study. With a sample size of 1000 participants, the expected halfwidth of the 95% confidence interval for the proportion of participants experiencing an SAE will be less than 0.05. The proportion of participants experiencing SAEs was calculated with 95% confidence intervals by the Clopper Pearson method. This analysis was repeated for the other two primary endpoints: the proportion of participants experiencing at least one AE within 14 days after the first vaccine dose and the proportion experiencing at least one AE within 28 days after the second dose. These summaries were repeated for Grade 3+ AEs, AEs starting on the vaccination date, and AEs starting within 3 days of vaccination. Prespecified subgroup analyses included testing for differences in the proportion of participants experiencing AEs on the day of vaccination and within 3 days of vaccination by sex (Fisher’s exact tests) and age categories (chi-squared tests). All analyses were performed using R version 4.3.3.

## Results

### Participants

500 participants enrolled from March 2023 to June 2024 and received a first dose of the vaccine (Supplementary Fig. S1). Of these initial 500 participants, 494 later received a second dose (99%). Adherence to the recommended vaccine schedule was very high—486 of the 494 second doses were within the protocol-specified window of 28-35 days after the first dose (98%). Most participants were male (391/500; 78%; Table 1). Ages ranged from 18 to 72 years (mean 34.4 years). Participants had very few reported comorbidities at baseline, the most common being hypertension (11/500; 2%).

**Table 1:** Study enrollment and baseline characteristics of the ITT population

Baseline Characteristics of the ITT Population (N = 500)	
Sex	
Male – *n* (%)	391 (78.2%)
Female – *n* (%)	109 (21.8%)
Age (years) – Mean (SD)	34.4 (11.4)
Weight (kg) – Mean (SD)	63.8 (15.3)
Height (cm) – Mean (SD)	169.7 (9.2)
**Comorbidities Present at Baseline –** *n* **(%)**	
Impaired immune function	0 (0%)
Lung disease	0 (0%)
Liver disease	0 (0%)
Neurologic disease or injury	0 (0%)
Psychiatric disease	0 (0%)
Cardiovascular disease	0 (0%)
Hypertension	11 (2.2%)
Kidney disease	0 (0%)
Diabetes	2 (0.4%)
Cancer	0 (0%)
HIV	0 (0%)
2 or more solicited comorbidities	0 (0%)

### Adverse events

There were no observed AEs of grade 3 or above and no observed SAEs during follow-up (Table 2). Of the 500 participants who received the first MVA-BN dose, 219 experienced at least one vaccination site AE within 14 days (44%; 95% CI 39% to 48%). Of the 494 who received the second dose, 102 experienced at least one vaccination site AE within 28 days (21%; 95% CI 17% to 24%). Overall, 35% of participants experienced at least one vaccination site AE (95% CI 31% to 39%).

**Table 2:** Proportion of participants experiencing adverse events and serious adverse events

Summary	Timing	Any Adverse Event n/N; prop. (95% CI)
At least one serious adverse event	Within 28 days of either dose	0/500; 0.00 (0.00, 0.01)
At least one adverse event of grade 3 or higher	Within 28 days of either dose	0/500; 0.00 (0.00, 0.01)
At least one adverse event of grade 2 or higher	Within 28 days of either dose	59/500; 0.12 (0.09, 0.15)
At least one adverse event (any grade)	Within 28 days of either dose	281/500; 0.56 (0.52, 0.61)
	On the day of vaccination (first dose)	80/500; 0.16 (0.13, 0.20)
	On the day of vaccination (second dose)	45/494; 0.09 (0.07, 0.12)
	On the day of vaccination (first or second dose)	111/500; 0.22 (0.19, 0.26)
	Within 3 days after vaccination (first or second dose)	241/500; 0.48 (0.44, 0.53)
	Within 14 days after the first vaccine dose	219/500; 0.44 (0.39, 0.48)
	Within 28 days after the second vaccine dose	102/494; 0.21 (0.17, 0.24)
At least one vaccination-site-reaction adverse event	Within 28 days of either dose	175/500; 0.35 (0.31, 0.39)
At least one non-vaccination-site-reaction adverse event	Within 28 days of either dose	192/500; 0.38 (0.34, 0.43)

Note that some categories overlap. For example, a participant experiencing an AE within 3 days of either dose also experienced an AE within 28 days of either dose.

**Fig. 1:**
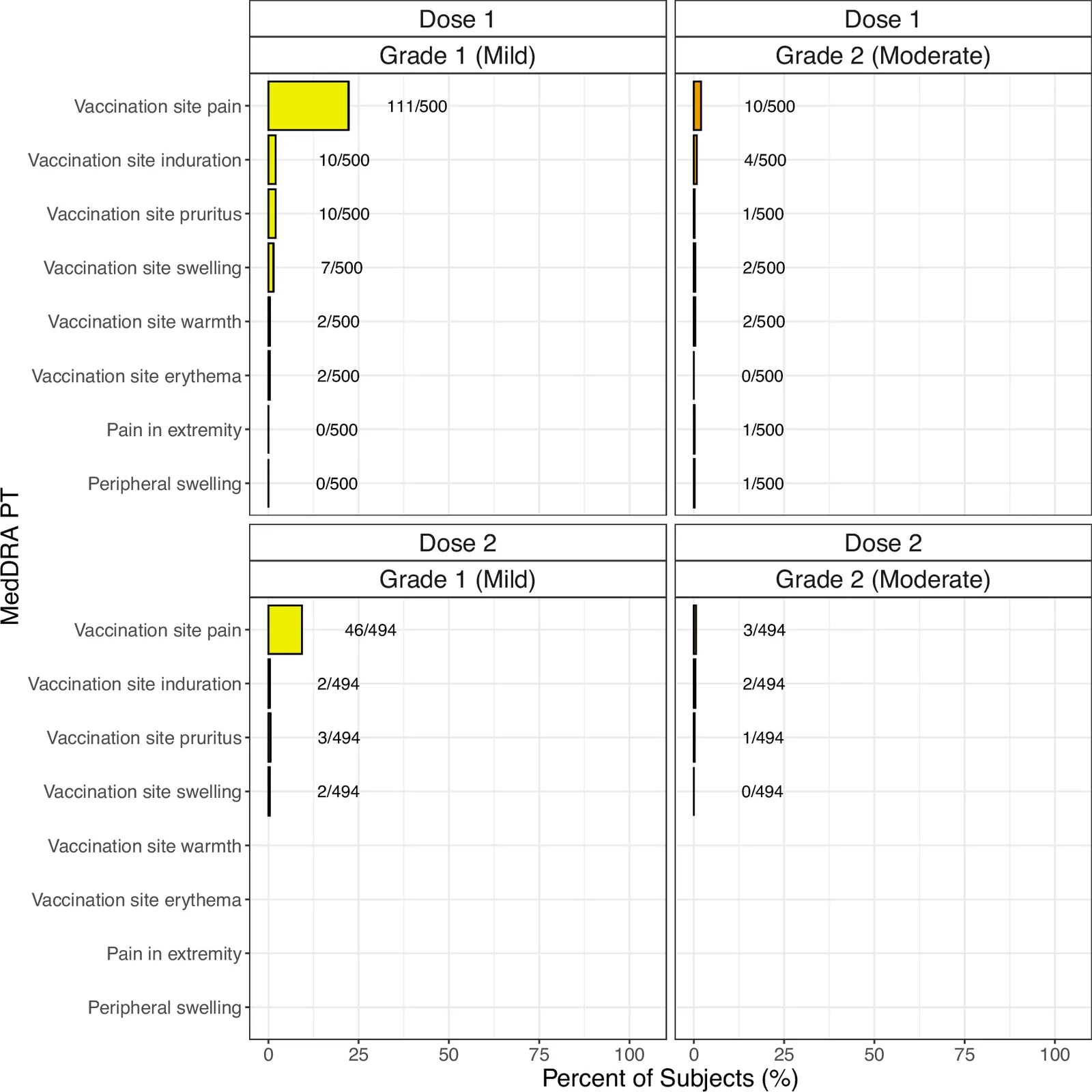
Summary of Vaccination Site Reaction Adverse Events – *N* (%) of Participants with Adverse Event by MedDRA Preferred Term and Dose. There were no Grade 3 or higher AEs. One reported vaccination site reaction AE (mild neck pain) was excluded due to an unknown day of onset, since it could not be determined whether the event began prior to Dose 1.

Although injection site reactions were fairly common, these reactions were generally mild (Fig. 1). For the first dose, 110/500 participants (22%) reported mild vaccination site pain. Less common AEs included moderate vaccination site pain (11/500; 2%), mild-to-moderate induration (14/500; 3%), itching/pruritus (11/500; 2%), and swelling (9/500; 2%). Vaccination site AEs reported for the second dose were less common and were similarly mild.

The percentage experiencing at least one AE on the day of vaccination was lower for males (80/391; 20%) than for females (31/109; 28%), although the difference was not statistically significant (*p* = 0.09; Table 3). There was no association between age and the likelihood of vaccination-related AEs.

**Table 3:** Subgroup analyses of the proportion of participants experiencing adverse events

Summary	Timing	Subgroup	Proportion (95% CI)	*P*-value^2^
At least one adverse event^1^	On the day of vaccination (first or second dose)	Males	80/391; 0.20 (0.17, 0.25)	0.09
		Females	31/109; 0.28 (0.20, 0.38)	
		Age 18-49	103/442; 0.23 (0.19, 0.28)	0.28
		Age 50-64	7/52; 0.13 (0.06, 0.26)	
		Age 65 and older	1/6; 0.17 (0.00, 0.64)	
	Within 3 days of vaccination (first or second dose)	Males	184/391; 0.47 (0.42, 0.52)	0.39
		Females	57/109; 0.52 (0.43, 0.62)	
		Age 18-49	213/442; 0.48 (0.43, 0.53)	0.99
		Age 50-64	25/52; 0.48 (0.34, 0.62)	
		Age 65 and older	3/6; 0.50 (0.12, 0.88)	

^1^All recorded adverse events were grade I (mild) or grade 2 (moderate).

^2^*P*-values testing for a difference in proportions between males and females are from Fisher’s exact test. P-values for differences by age group are from a permuted chi-squared test.

Among non-injection-site-reaction AEs, the most common were mild or moderate headache (14% of participants) and mild or moderate pyrexia/fever (11%; Table 4). All other non-injection-site-reaction AEs occurred in ≤4% of participants.

Hypoesthesia (loss of sensation) was reported in 1 participant (0.2%).

**Table 4:** Non-vaccination-site-reaction adverse events within 28 days of Dose 1 or Dose 2 by MedDRA Preferred Term (PT), Grade, and Frequency

MedDRA preferred term (PT)	Number and percentage (%) of participants with at least one non-vaccination-site-reaction AE. Denominators include all 500 participants.		
	Any grade^1^	Grade 1	Grade 2
Headache	69 (13.8)	59 (11.8)	11 (2.2)
Pyrexia	57 (11.4)	48 (9.6)	11 (2.2)
Cough	18 (3.6)	13 (2.6)	5 (1.0)
Pruritus	16 (3.2)	12 (2.4)	4 (0.8)
Chills	11 (2.2)	11 (2.2)	0 (0)
Asthenia	10 (2.0)	10 (2.0)	0 (0)
Diarrhoea	10 (2.0)	9 (1.8)	1 (0.2)
Vertigo	9 (1.8)	8 (1.6)	1 (0.2)
Dyspepsia	8 (1.6)	5 (1.0)	4 (0.8)
Back pain	7 (1.4)	6 (1.2)	1 (0.2)
Myalgia	7 (1.4)	7 (1.4)	0 (0)
Arthralgia	6 (1.2)	6 (1.2)	0 (0)
Nausea	6 (1.2)	6 (1.2)	0 (0)
Abdominal pain	5 (1.0)	5 (1.0)	0 (0)
Abdominal pain upper	5 (1.0)	5 (1.0)	0 (0)
Influenza	5 (1.0)	4 (0.8)	2 (0.4)
Limb discomfort	5 (1.0)	5 (1.0)	0 (0)
Neck pain	5 (1.0)	4 (0.8)	1 (0.2)
Dysphagia	4 (0.8)	3 (0.6)	1 (0.2)
Nasopharyngitis	4 (0.8)	1 (0.2)	3 (0.6)
Productive cough	4 (0.8)	3 (0.6)	1 (0.2)
Somnolence	4 (0.8)	4 (0.8)	0 (0)
Pain	3 (0.6)	3 (0.6)	0 (0)
Abdominal distension	2 (0.4)	2 (0.4)	0 (0)
Lymphadenopathy	2 (0.4)	2 (0.4)	0 (0)
Pain in extremity	2 (0.4)	0 (0)	2 (0.4)
Rash	2 (0.4)	2 (0.4)	0 (0)
Chest pain	1 (0.2)	0 (0)	1 (0.2)
Choking	1 (0.2)	1 (0.2)	0 (0)
Decreased appetite	1 (0.2)	1 (0.2)	0 (0)
Dental caries	1 (0.2)	0 (0)	1 (0.2)
Dental discomfort	1 (0.2)	0 (0)	1 (0.2)
Dizziness	1 (0.2)	1 (0.2)	0 (0)
Dysuria	1 (0.2)	1 (0.2)	0 (0)
Epistaxis	1 (0.2)	1 (0.2)	0 (0)
Eye pain	1 (0.2)	1 (0.2)	0 (0)
Eye pruritus	1 (0.2)	1 (0.2)	0 (0)
Hypoaesthesia	1 (0.2)	0 (0)	1 (0.2)
Maxillary expansion	1 (0.2)	0 (0)	1 (0.2)
Pelvic pain	1 (0.2)	0 (0)	1 (0.2)
Post-traumatic pain	1 (0.2)	0 (0)	1 (0.2)
Rash vesicular	1 (0.2)	0 (0)	1 (0.2)
Rhinorrhoea	1 (0.2)	1 (0.2)	0 (0)
Subcutaneous abscess	1 (0.2)	0 (0)	1 (0.2)
Urticaria	1 (0.2)	0 (0)	1 (0.2)
Vision blurred	1 (0.2)	1 (0.2)	0 (0)
Vulvovaginal pruritus	1 (0.2)	0 (0)	1 (0.2)

^1^No AEs of grade 3 or higher were observed in the study. Individuals may have had separate grade 1 and grade 2 events, so the total number with any grade may be lower than the sum of participants with grade I and participants with grade II events. Every AE was classified as a vaccination-site-reaction AE or not a vaccination-site-reaction AE.

### Breakthrough Infection

One breakthrough MPXV infection was reported for one participant who completed the full study protocol, but who developed monkeypox approximately 3 months after the second vaccine dose. This participant was a nurse for the connected monkeypox RCT and thus more exposed to monkeypox patients than many other participants. The infection was mild and resolved within seven days following standard supportive treatment. The participant was not immunocompromised, adhered to all infection prevention and control measures, and did not have any known exposure to monkeypox outside nursing activities.

## Pregnancies

A total of four participants had a positive pregnancy test during follow-up, after receiving the first vaccine dose. In accordance with the protocol, they did not receive the second dose, but attempts were made to record the eventual pregnancy outcomes. Two declined to provide pregnancy outcome data. Of the remaining two, one resulted in a live birth at 39 weeks with no detected complications, and the other ended in miscarriage during the third month. The miscarriage was considered not related to the administration of the vaccine.

## Discussion

This study was the second to evaluate safety in a large cohort of adult MVA-BN monkeypox vaccine recipients in the DRC. Consistent with previous studies, our observations suggest that the vaccine is safe for use. Injection site reactions, including pain, itching, and swelling, were common but were typically mild. No AEs of grade 3 or higher were observed in our study.

The study enrolled personnel and associated staff of PALM 007, a randomized, placebo-controlled trial of tecovirimat. This population was comprised of generally healthy, employed adults with few comorbidities and good nutritional status. This population is not necessarily representative of the group most at-risk for monkeypox in DRC, where the disease typically occurs in rural communities with higher malnutrition rates^24,25^ and limited access to healthcare, and disproportionately impacts children. Participants were 78.2% male, reflecting the demographic composition of the healthcare personnel at the recruitment sites.

Minhaj et al. recently reported safety results from a similar study to our own^16^. They enrolled 1600 healthcare workers, primarily in the Tshuapa province of DRC, with 1000 receiving a liquid formulation of MVA-BN and 600 receiving a reconstituted lyophilized formulation. They observed no significant difference in the risk of AEs between formulations and noted an overall proportion with local injection-site reactions (36%) that nearly matches the result from our study (35%). Neither study observed any SAEs related to vaccine administration. Both our results and those of Minhaj et al. align with previous studies conducted in non-endemic areas^17–19,26,27^ and a meta-analysis comparing SAE frequency between MVA-BN and placebo recipients^28^. The long-sought availability of safety results from two high-quality trials carried out in DRC reflects an increasing awareness of the value of conducting clinical research in the areas most impacted by disease, and their close alignment with older studies provides a positive public health message that can be used to improve vaccine trust in DRC.

Other than injection site reactions, the most common other AEs were headache (14%) and fever (13.4%). This is consistent with other studies that have also identified mild headache as a common side effect the MVA-BN vaccine^13,17–19,26–28^. Of the other AEs recorded, each affected less than 5% of participants.

The distribution of AEs was comparable across age groups and sex. The proportion with at least one AE on the day of vaccination was slightly higher for females than males (28% vs. 20%). The difference is not statistically significant but is consistent with other studies that have shown a higher frequency of AEs in women compared to men^29,30^.

Most AEs occurred within the first 3 days after receiving a dose, underlining the need to be attentive to the recipient on the day of vaccination and the following few days. Fewer AEs were noted after the second dose compared to the first. This trend was also observed in a meta-analysis and may be due to the non-replicative nature of MVA-BN, which limits cumulative reactogenicity compared to replicating vaccines^28^. It may also be due to adaptation, whether physiological or even psychological, to the administration of the vaccine.

Breakthrough infection has been reported after vaccination with MVA-BN, but clinical symptoms and signs tend to be milder^31–34^. The US Center for Disease Control (CDC) estimates that this occurs in less than 1% of individuals who completed the 2-dose regimen^35^, which aligns with our observation. The lone breakthrough case was mild, but this study was not designed to collect additional clinical data for further disease characterization. The DRC Ethics Committee did permit the gathering of prior monkeypox exposure information from this participant, but lesion counts and serological data could not be obtained. No additional exposure was identified beyond the participant’s role as a nurse in the PALM 007 monkeypox RCT.

This study did not include a control arm, so comparable rates of events in unvaccinated individuals could not be assessed. The causal relationship of AEs to vaccine receipt was also not directly assessed by clinicians; however, the immediate post-injection reactions may be assumed to be related to the vaccination. All other events are reported for transparency, regardless of their potential causality. Most events were mild, aligning with previous studies and suggesting that the vaccine is generally safe for healthy adults in DRC. EKGs were not performed, and thus, we are unable to rule out certain cardiac adverse events of special interest (AESI). We recommend randomized controlled trials whenever they are logistically and ethically possible to provide the highest quality of evidence regarding vaccine safety.

The sample size of 500 precludes detection of very rare, but possibly related, AEs. Hypoesthesia was observed in one participant in this study (0.2%). A previous observational cohort study analyzed ten AESI, including cardiac AESI, in over 50,000 U.S. adults who received MVA-BN between May 2022 and March 2023 in the Vaccine Safety Datalink (VSD)^36^. Observed 28-day post-vaccination standardized incidence rates were compared to expected rates for each AESI, and none differed significantly. The authors of that study did not rule out the possibility of rare vaccine-associated AEs, but we can expect them to be extremely rare if an association exists. It may be important to continue monitoring the occurrence of rare AEs in future studies.

Acknowledging these limitations, this evaluation of the safety of the MVA-BN vaccine in a Congolese adult population confirms that vaccination is generally well-tolerated, with mild AEs consistent with what has been reported by previous studies in non-endemic areas. Vaccine safety remains to be evaluated in special populations, including children, pregnant or breastfeeding women, and the immunocompromised. Large, randomized, controlled studies conducted in the most at-risk areas should be prioritized to further establish the safety and efficacy profile of the MVA-BN vaccine.

## Supplementary information

Supplementary Information

## Data Availability

De-identified participant data sufficient to allow reproduction of the key results will be made available for request through the AccessClinicalData@NIAID data sharing platform, a secure, cloud-based data sharing platform that provides access to datasets from NIAID-supported trials. A data dictionary will be included with shared data. Data access requests are subject to approval by NIAID.

## References

[1] Bangwen, E. et al. Suspected and confirmed monkeypox cases in DR Congo: a retrospective analysis of national epidemiological and laboratory surveillance data, 2010–23. *Lancet* **405**, 408–419 (2025). 39892912 10.1016/S0140-6736(24)02669-2 PMC7618260

[2] US Centers for Disease Control and Prevention. How Monkeypox Spreads. https://www.cdc.gov/monkeypox/causes/index.html (accessed April 24 2026).

[3] Isidro, J. et al. Phylogenomic characterization and signs of microevolution in the 2022 multi-country outbreak of monkeypox virus. *Nat. Med.* **28**, 1569–1572 (2022). 35750157 10.1038/s41591-022-01907-y PMC9388373

[4] European Centre for Disease Prevention and Control. Monkeypox. https://www.ecdc.europa.eu/en/monkeypox (accessed April 24 2026).

[5] Thornhill, J. P. et al. Monkeypox virus infection in humans across 16 countries — April–June 2022. *N. Engl. J. Med.* **387**, 679–691 (2022). 35866746 10.1056/NEJMoa2207323

[6] Gigante, C. M. et al. Multiple lineages of monkeypox virus detected in the United States, 2021-2022. *Science* **378**, 560–565 (2022). 36264825 10.1126/science.add4153 PMC10258808

[7] De Vos, E. et al. Potential determinants of the decline in monkeypox cases in Belgium: A behavioral, epidemiological and seroprevalence study. *Int. J. Infect. Dis.* **146**, 107132 (2024). 10.1016/j.ijid.2024.107132 38942168

[8] Pekar, J. E. et al. Transmission dynamics of the 2022 monkeypox epidemic in New York City. *Nat. Med.* **31**, 1464–1473 (2025). 40133528 10.1038/s41591-025-03526-9 PMC12092259

[9] Brosius, I. et al. Epidemiological and clinical features of monkeypox during the clade Ib outbreak in South Kivu, Democratic Republic of the Congo: a prospective cohort study. *Lancet* **405**, 547–559 (2025). 39892407 10.1016/S0140-6736(25)00047-9 PMC7618259

[10] World Health Organization. WHO Director-General declares monkeypox outbreak a public health emergency of international concern. https://www.who.int/news/item/14-08-2024-who-director-general-declares-monkeypox-outbreak-a-public-health-emergency-of-international-concern (accessed April 24, 2026).

[11] Makangara-Cigolo, J. C. et al. Clade Ia Monkeypox virus linked to sexual transmission, Democratic Republic of the Congo, August 2024. *Emerg. Infect. Dis.* **31**, 1033–1034 (2025). 40263113 10.3201/eid3105.241690 PMC12044235

[12] Vakaniaki, E. H. et al. Sustained human outbreak of a new MPXV clade I lineage in eastern Democratic Republic of the Congo. *Nat. Med.* **30**, 2791–2795 (2024). 38871006 10.1038/s41591-024-03130-3 PMC11485229

[13] U.S. Food and Drug Administration. JYNNEOS. https://www.fda.gov/vaccines-blood-biologics/jynneos (accessed April 24 2026).

[14] U.S. Centers for Disease Control and Prevention. Considerations for Intradermal Administration of JYNNEOS Vaccine. https://www.cdc.gov/monkeypox/hcp/vaccine-considerations/intradermal-administration.html (accessed April 24 2026).

[15] Priyamvada, L. et al. Serological responses to the MVA-based JYNNEOS monkeypox vaccine in a cohort of participants from the Democratic Republic of Congo. *Vaccine* **40**, 7321–7327 (2022). 36344361 10.1016/j.vaccine.2022.10.078 PMC9635871

[16] Minhaj, F. S. et al. Safety of MVA-BN vaccine in health-care personnel in DR Congo: a prospective cohort study. *Lancet Infect. Dis.* (2026). 10.1016/S1473-3099(25)00779-0 41819117

[17] Back, S. et al. Effectiveness and safety of the MVA–BN vaccine against monkeypox in at-risk individuals in the United States (USMVAc). *Vaccines* **12** 10.3390/vaccines12060651 PMC11209565 38932380

[18] Hillus, D. et al. Safety and effectiveness of MVA-BN vaccination against monkeypox in at-risk individuals in Germany (SEMVAc and TEMVAc): a combined prospective and retrospective cohort study. *Lancet Infect. Dis.* **25**, 775–787 (2025). 40118087 10.1016/S1473-3099(25)00018-0

[19] Sah, R. et al. Monkeypox (Monkeypox) vaccines and their side effects: the other side of the coin. *Int J. Surg.* **109**, 215–217 (2023). 36799858 10.1097/JS9.0000000000000142 PMC10389550

[20] U.S. Department of Health and Human Services. Intradermal JYNNEOS Monkeypox Vaccine Fast Facts. FDA. https://www.fda.gov/media/161051/download (accessed April 24 2026).

[21] European Medicines Agency (EMA). EMA recommends approval of Imvanex for the prevention of monkeypox disease. https://www.ema.europa.eu/en/news/ema-recommends-approval-imvanex-prevention-monkeypox-disease (accessed April 24 2026).

[22] U.S. Department of Health and Human Services. Common Terminology Criteria for Adverse Events (CTCAE) Version 5.0. National Cancer Institute https://dctd.cancer.gov/research/ctep-trials/for-sites/adverse-events/ctcae-v5-5x7.pdf (accessed April 24 2026). (2017).

[23] International Council for Harmonisation of Technical Requirements for Pharmaceuticals for Human Use. Clinical Safety Data Management: Definitions and Standards for Expedited Reporting E2A. Published October 27, 1994. https://database.ich.org/sites/default/files/E2A_Guideline.pdf (accessed April 24 2026).

[24] Global Nutrition Report | Country Nutrition Profiles - Global Nutrition Report n.d. https://globalnutritionreport.org/resources/nutrition-profiles/africa/middle-africa/democratic-republic-congo/ (accessed April 24, 2026).

[25] Likaka, E., Kiangana, E. & Ngaboyeka, G. Access of Households to Arable Land and Nutritional Status of Children Aged 6–59 Months in Rural Areas of South Kivu, Case of the Health Zone of Minova, Eastern DRC. Rural Health - Investment, Research and Implications, IntechOpen

[26] Deng, L. et al. Short-term adverse events following immunization with modified Vaccinia Ankara-Bavarian Nordic (MVA-BN) Vaccine for Monkeypox. *JAMA* **329**, 2091–2094 (2023). 10.1001/jama.2023.7683 PMC10282881 37145654

[27] Overton, E. T. et al. A randomized phase II trial to compare safety and immunogenicity of the MVA-BN smallpox vaccine at various doses in adults with a history of AIDS. *Vaccine* **38**, 2600–2607 (2020). 32057574 10.1016/j.vaccine.2020.01.058

[28] Nave, L. et al. Immunogenicity and Safety of Modified Vaccinia Ankara (MVA) Vaccine—a systematic review and meta-analysis of randomized controlled trials. *Vaccines* **11**, 1410 (2023). 10.3390/vaccines11091410 PMC10536351 37766090

[29] Watson, S., Caster, O., Rochon, P. A. & den Ruijter, H. Reported adverse drug reactions in women and men: Aggregated evidence from globally collected individual case reports during half a century. *EClinicalMedicine* **17**, (2019). 10.1016/j.eclinm.2019.10.001 PMC6933269 31891132

[30] Zucker, I. & Prendergast, BJ. Sex differences in pharmacokinetics predict adverse drug reactions in women. *Biol Sex Differ* **11**, 32 (2020). 32503637 10.1186/s13293-020-00308-5 PMC7275616

[31] Liu, W. D., Chao, T. L., Chang, S. Y. & Hung, C. C. Serum Mpox-specific IgG titers before and after breakthrough Mpox infection in an HIV-infected individual with viral suppression and prior 2-dose Mpox vaccination. *J. Microbiol. Immunol. Infect.* **58**, 149–151 (2025). 39472243 10.1016/j.jmii.2024.10.003

[32] Hazra, A. et al. Monkeypox in people with past infection or a complete vaccination course: a global case series. *Lancet Infect. Dis.* **24**, 57–64 (2024). 37678309 10.1016/S1473-3099(23)00492-9

[33] Bottanelli, M. et al. A case of breakthrough monkeypox infection in an individual non-responder to MVA-BN vaccination. *Lancet Infect. Dis.* **24**, e11–e12 (2024). 38071992 10.1016/S1473-3099(23)00741-7

[34] Cornelisse, V. J. et al. Case study: breakthrough monkeypox infection in Aotearoa New Zealand and Australia after completed two-dose course of subcutaneous modified vaccinia Ankara (MVA-BN) vaccines. *Sex. Health* **20**, 585–587 (2023). 37852607 10.1071/SH23139

[35] Guagliardo, S. A. et al. Monkeypox virus infections after 2 preexposure doses of JYNNEOS vaccine — United States, May 2022–May 2024. *MMWR Morb. Mortal. Wkly Rep.* **73**, 460–466 (2024). 38781111 10.15585/mmwr.mm7320a3 PMC11115437

[36] Duffy, J. et al. JYNNEOS vaccine safety surveillance in the Vaccine Safety Datalink during the 2022 mpox outbreak in the United States. *Infection* **53**, 1321–1328 (2024). 39565485 10.1007/s15010-024-02428-1 PMC12089422

